# Supplementary material for: Drug Metabolite Cluster-Based Data-Mining Method for Comprehensive Metabolism Study of 5-hydroxy-6,7,3′,4′-tetramethoxyflavone in Rats
Source: Molecules. 2019 Sep 9;24(18):3278. doi: 10.3390/molecules24183278 (PMC6767304; doi:10.3390/molecules24183278)
Supplement: Supplementary file 1 [file molecules-24-03278-s001.pdf]

Supplementary Materials:

Captions

Fig. S1. ESI-MS^n spectra of monohydroxyl-tetramethoxyflavones **(M0, M9-M10)** in positive ion mode.

(A)MS^2 spectrum of **M0**; (B) MS^3 spectrum of **M0**;

(C) MS^2 spectrum of **M9**; (D) MS^2 spectrum of **M10**

Fig. S2. ESI-MS^n spectra of dihydroxyl-triamethoxyflavone (**M5-M7**) in negative ion mode.

(A)MS^2 spectrum of **M5**; (B) MS^3 spectrum of **M5**;

(C) MS^2 spectrum of **M6**; (D) MS^2 spectrum of **M7**

Fig. S3. ESI-MS^n spectra of monohydroxyl-tetramethoxyflavanones (**M14-M15**) in positive ion.

(A)MS^2 spectrum of **M14**; (B) MS^3 spectrum of **M14**;

(C) MS^2 spectrum of **M15**; (D) MS^3 spectrum of **M15**

Fig. S4. ESI-MS^n spectra of dihydroxyl- tetramethoxychalcone (**M34-M37**) in positive ion mode.

(A) MS^2 spectrum of **M34**; (B) MS^2 spectrum of **M35**;

(C) MS^2 spectrum of **M36**; (D) MS^2 spectrum of **M37**

Fig. S5. ESI-MS^n spectra of conjugate-metablites (**M72, M78**) in negative ion mode.

(A) MS^2 spectrum of **M72**; (B) MS^3 spectrum of **M72**;

(C) MS^2 spectrum of **M78**; (D) MS^3 spectrum of **M78**

Table S1. Summary of HTF metabolites numbers of substituent groups in rats urine and plasma.

Table S2. Template for searching for these metabolites and their derivatives from metabolic reactions.

Table S3. Summary of HTF metabolites based on DMC

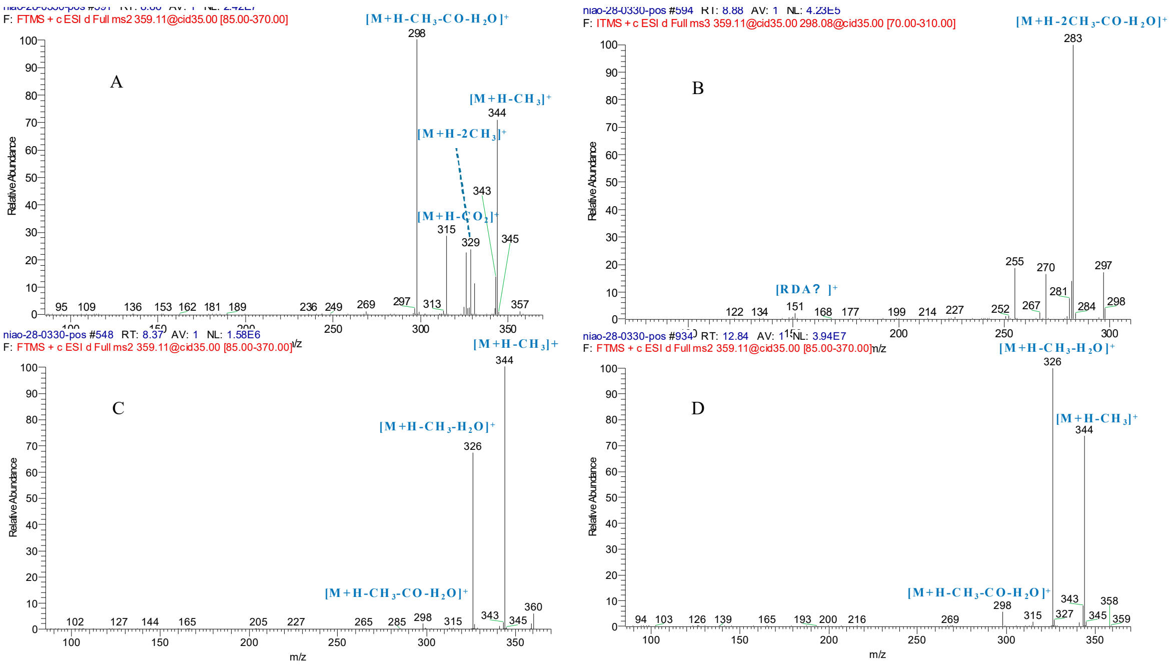

Fig.S1. ESI-MS^n spectra of monohydroxyl-tetramethoxyflavones **(M0, M9-M10)** in positive ion mode.

(A)MS^2 spectrum of **M0**; (B) MS^3 spectrum of **M0**;

(C) MS^2 spectrum of **M9**; (D) MS^2 spectrum of **M10**

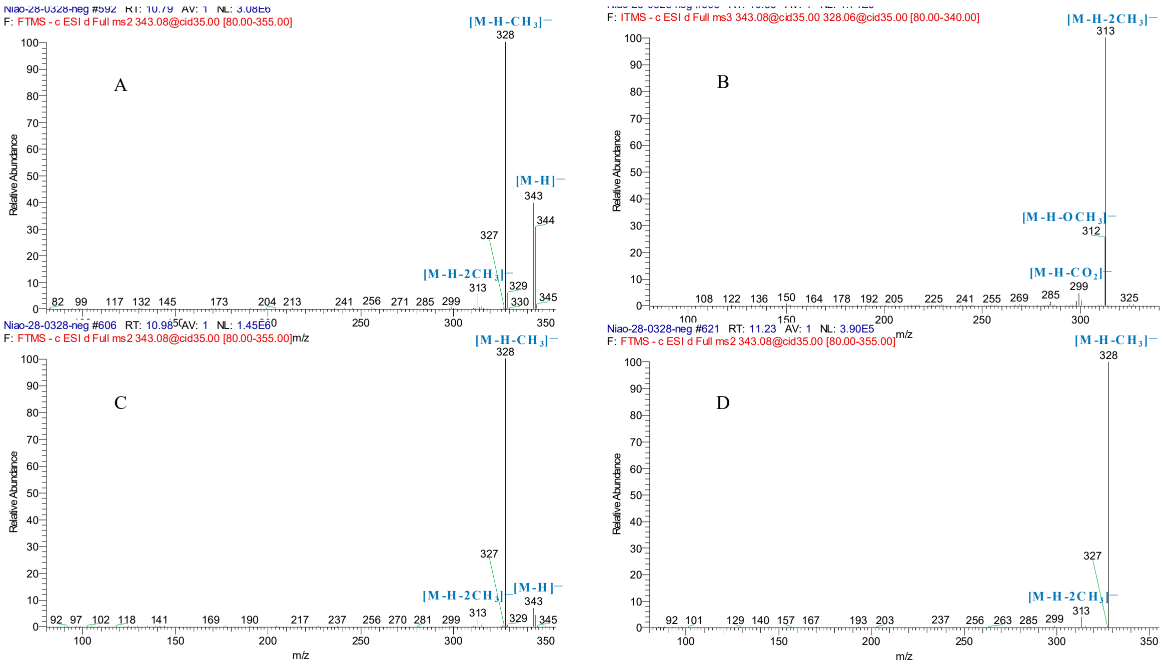

Fig. S2. ESI-MS^n spectra of dihydroxyl-triamethoxyflavone (**M5-M7**) in negative ion mode.

(A)MS^2 spectrum of **M5**; (B) MS^3 spectrum of **M5**;

(C) MS^2 spectrum of **M6**; (D) MS^2 spectrum of **M7**

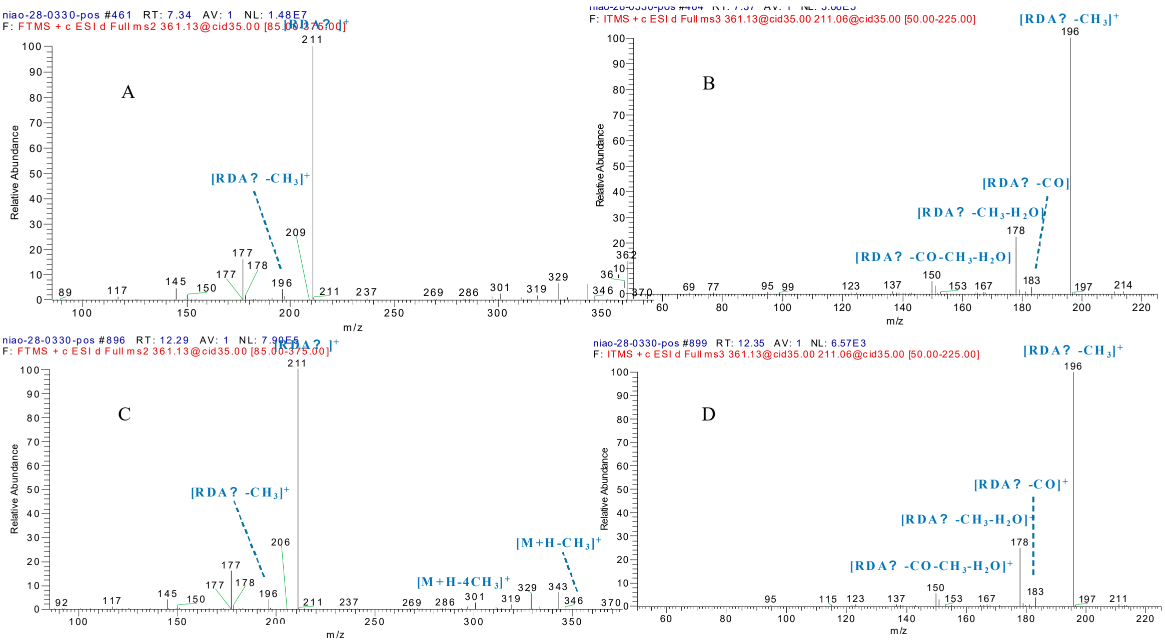

Fig. S3. ESI-MS^n spectra of monohydroxyl-tetramethoxyflavanones (**M14**-**M15**) in positive ion (A)MS^2 spectrum of **M14**; (B) MS^3 spectrum of **M14**; (C) MS^2 spectrum of **M15**; (D) MS^3 spectrum of **M15**

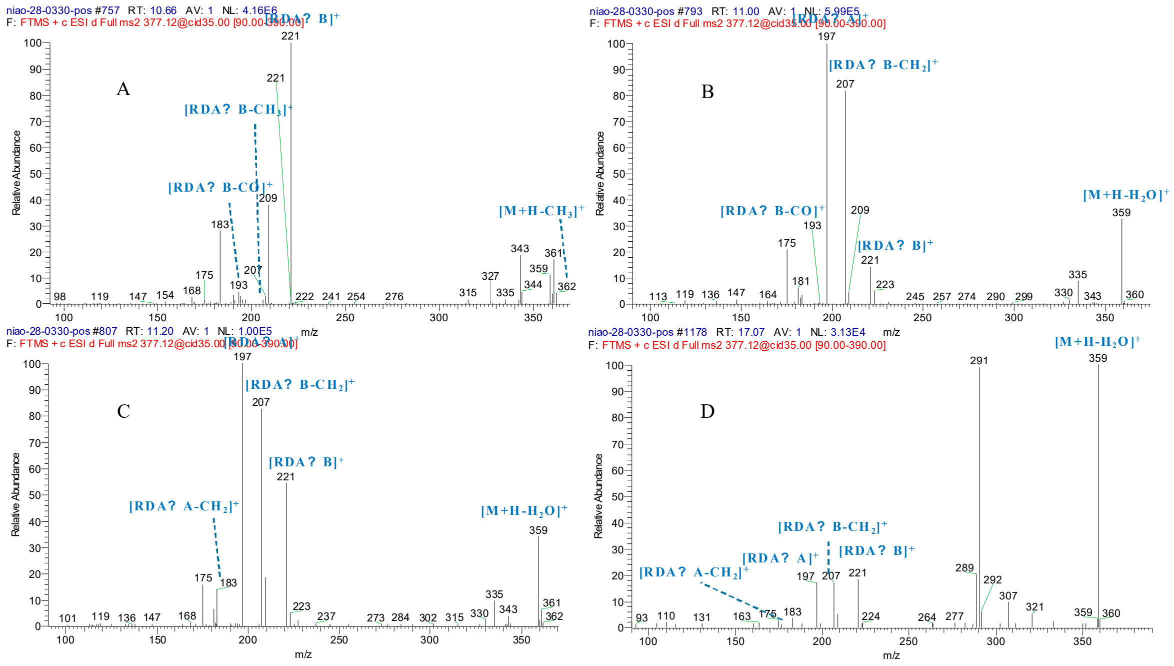

Fig. S4. ESI-MS^n spectra of dihydroxyl- tetramethoxychalcone (**M34-M37**) in positive ion mode.

(A) MS^2 spectrum of **M34**; (B) MS^2 spectrum of **M35**;

(C) MS^2 spectrum of **M36**; (D) MS^2 spectrum of **M37**

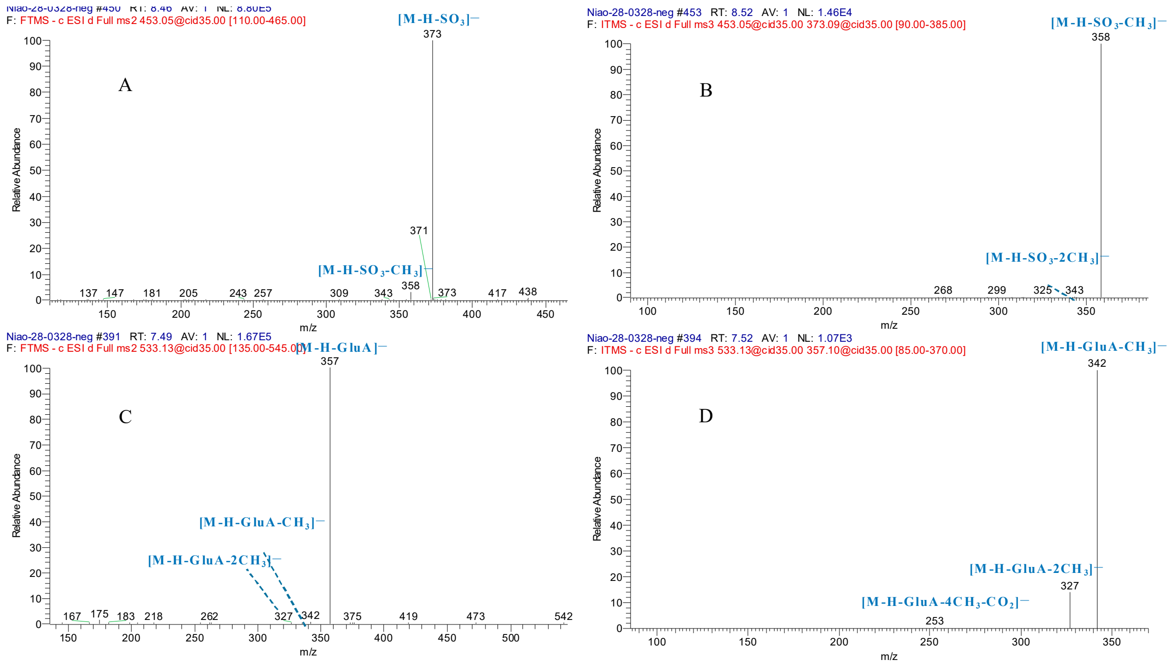

Fig. S5. ESI-MS^n spectra of conjugate-metablites (**M72**, **M78**) in negative ion mode. (A) MS^2 spectrum of **M72**; (B) MS^3 spectrum of **M72**; (C) MS^2 spectrum of **M78**; (D) MS^3 spectrum of **M78**

Table S1. Summary of HTF metabolites numbers of substituent groups in rats urine and plasma.

Peak	-OH	$-OCH_3$	Urine	Plasma	Peak	-OH	$-OCH_3$	Urine	Plasma
**M0**	1	4	+	+	**M44**	1	5	-	+
**M1**	3	2	+	+	**M45**	1	5	+	+
**M2**	3	2	+	-	**M46**	1	5	-	+
**M3**	3	2	+	+	**M47**	1	5	-	+
**M4**	3	2	-	+	**M48**	1	5	-	+
**M5**	2	3	+	+	**M49**	1	5	-	+
**M6**	2	3	+	+	**M50**	1	5	-	+
**M7**	2	3	+	-	**M51**	1	5	+	+
**M8**	2	3	-	+	**M52**	1	5	+	+
**M9**	1	4	+	+	**M53**	1	5	+	+
**M10**	1	4	+	+	**M54**	1	5	-	+
**M11**	3	3	-	+	**M55**	1	5	+	+
**M12**	1	5	+	+	**M56**	1	5	+	+
**M13**	1	5	+	-	**M57**	1	5	+	+
**M14**	1	4	+	+	**M58**	1	5	+	+
**M15**	1	4	-	+	**M59**	1	5	+	+
**M16**	3	3	-	+	**M60**	1	5	-	+
**M17**	3	3	-	+	**M61**	1	6	+	+

**M18**	3	3	-	+	**M62**	1	6	-	+
**M19**	3	3	-	+	**M63**	1	6	+	+
**M20**	0	5	+	+	**M64**	1	6	-	+
**M21**	0	5	+	-	**M65**	1	6	-	+
**M22**	0	5	+	-	**M66**	3	4	+	+
**M23**	0	5	-	+	**M67**	3	4	+	+
**M24**	2	4	-	+	**M68**	3	4	+	+
**M25**	2	4	-	+	**M69**	0	6	+	+
**M26**	2	4	-	+	**M70**	0	6	+	+
**M27**	2	4	+	+	**M71**	0	6	+	-
**M28**	2	4	-	+	**M72**	0	4	-	+
**M29**	2	4	-	+	**M73**	0	5	+	+
**M30**	2	4	-	+	**M74**	0	5	+	+
**M31**	0	5	-	+	**M75**	0	5	+	+
**M32**	2	4	+	+	**M76**	0	5	-	+
**M33**	2	4	+	+	**M77**	1	3	-	+
**M34**	2	4	+	+	**M78**	0	4	+	+
**M35**	2	4	-	+	**M79**	0	4	+	+
**M36**	2	4	-	+	**M80**	1	4	-	+
**M37**	2	4	-	+	**M81**	1	4	-	+
**M38**	1	5	+	+	**M82**	1	4	+	+
**M39**	1	5	+	+	**M83**	0	5	+	+

**M40**	1	5	+	+	**M84**	0	5	+	+
**M41**	1	5	+	+	**M85**	0	5	+	+
**M42**	1	5	+	-	**M86**	0	5	+	+
**M43**	1	5	+	+					

molecules MDPI

Table S2. Template for searching for these metabolites and their derivatives from metabolic reactions.

Biotransformation reaction	Molecular formula change	Mass Shift
R-$CH_2C_6H_5$→R-H	-C_7H_6	-90.0468
R-^{79}B→R-H	-Br+H	-77.9104
R-CF_3→R-H	-CF_3+H	-67.9874
2R-$^{35}Cl_2$→R-H	-Cl_2+H_2	-67.9222
R-^{79}Br→R-OH	-Br+OH	-61.9156
R-C(CH_3)$_3$→R-H	-C_4H_8	-56.0624
R-ONO_2→R-OH	-NO_2+H	-44.9851
R-COOH→R-H	-CO_2	-43.9898
R-CH(CH_3)$_2$→R-H	-C_3H_6	-42.0468
R-C(CH_3)$_3$→R-OH	-C_4H_8+O	-40.0675
2R-F_2→R-H	-F_2+H_2	-35.9811
R-^{35}Cl→R-H	-Cl+H	-33.9611
R-CH_2OH→R-H	-CH_2O	-30.0106
R-NO_2→R-NH_2	-O_2+H_2	-29.9742
R-$CH_2OCH_2CH_2CH_3$→R-COOH	-C_3H_8+O	-28.0675
R-C_2H_5→R-H	-C_2H_4	-28.0312
R-CO-R'→R-R'	-CO	-27.9949
R-CH(CH_3)$_2$→R-OH	-C_3H_6+O	-26.0519
R-CH=N-OH→R-CN	-H_2O	-18.0105
R-F→R-H	-F+H	-17.9906
R-^{35}Cl→R-OH	-Cl+OH	-17.9662
RR'S=O→R-S-R'	-O	-15.9949
R-NHNHR'C=S→R-NHNHR'C=O	-S+O	-15.9772
R-$CH_2OCH_2CH_3$→R-COOH	-C_2H_6+O	-14.0519
R-CH_3→R-H	-CH_2	-14.0157
R-C(CH_3)$_3$→R-COOH	-C_3H_8+O_2	-12.0726
R-CH_2CH_3→R-OH	-C_2H_4+O	-12.0363

R-CH_2-CH_2-CH_2-CH_2-R'→R-CH=CH-CH=CH-R'	-H_4	-4.0314
R-F→R-OH	-F+OH	-1.9957
R-$CHNH_2$-R'→R-CO-R'	-NH_3+O	-1.0316
R-CH_2OCH_3→R-COOH	-CH_4+O	-0.0365
R-CH_2-NH_2→R-CH_2-OH	-NH+O	+0.984
R-CH(CH_3)$_2$→R-COOH	-C_2H_6+O_2	+1.943
R-CH_3→R-OH	-CH_2+O	+1.9792
R-CO-R'→R-CHOH-R'	+H_2	+2.0157
R-CH_2-R'→R-C(O)-R'	-H_2+O	+13.9792
R-XH→R-X-CH_2(X=N,O,S)	+CH_2	+15.9586
R-CH_2CH_3→R-COOH	-CH_4+O_2	+15.9949
R-CH_2CH_2→R-CH-(OH)$_2$	-CH_2+O_2	+17.9741
R-CH=CH-R→R-CH_2-CHOH-R'	+H_2O	+18.0106
R-H→R-O-CH_3	+CH_2O	+30.0105
R-CH=CH-R'→R-CH(OH)-CH(OH)-R'	+H_2O_2	+34.0054
R-NH_2→R-$NHCOCH_3$	+C_2H_2O	+42.0106
R-COOH→R-$CONHCH_2COOH$	+C_2H_3NO	+57.0215
R-OH→R-OSO_3H	+SO_3	+79.9568
R-H→R-OSO_3H	+SO_4	+95.9517
R-COOH→R-CONH-CH(CH_2SH)-COOH	+C_3H_5NOS	+103.0092
R-COOH→R-CONH-$CH_2CH_2SO_3H$	+$C_2H_5NO_2S$	+107.0041
R-CH_2-R'→RR'-CH-SCH_2CH(NH_2)-COOH	+$C_3H_5NO_2S$	+119.0041
R-COOH→R-CO-SCH_2CH($NHCOCH_3$)COOH-CO	+$C_5H_7NO_2S$	+145.0198
R-COOH→R-$C_6H_8O_6$	+$C_5H_8O_5$	+148.0372
RR'-CH_2→RR'-CH-SCH_2CH($NHCOCH_3$)-COOH	+$C_5H_7NO_3S$	+161.0147
R-OH→R-O-C_6H11O_5	+$C_6H_{10}O_5$	+162.0528
R-OH→R-O-$C_6H_9O_6$	+$C_6H_8O_6$	+176.0321
R-H→R-O-$C_6H_9O_6$	+$C_6H_8O_7$	+192.0270
R-COOH→R-CO-GSH	+C_{10}H1$_5N_3O_6$S	+305.6820

Table S3. Summary of HTF metabolites based on DMC

Metabolites Cluster	Identification/Reactions	Metabolites Number
Prototype drug	5-hydroxy-6,7,3',4'-tetramethoxyflavone	M0
Prototype drug isomer	5-hydroxy-6,7,3',4'-tetramethoxyflavone	M9-M10
Primarry cluster	Demethylation	M5-M7
	Flavanone Formation	M14-M15
	Sulfate-Conjugation	M72
	Methylation	M20-M23
	Methoxylation	M38-M50
	Glucuronide Conjugation	M78
	Oxidation	M24-M30
Secondary cluster	Demethylation<br>Oxidation	M11
	Di-demethylation	M1-M4
	Flavanone Formation<br>Demethylation	M8
	Flavanone Formation<br>Oxidation	M32-M33
	Flavanone Formation<br>Di-Oxidation	M67-M68
	Flavanone Formation<br>Methylation	M31
	Flavanone Formation<br>Methoxylation	M51-M53
	Sulfate Conjugation<br>Methoxylation	M73
	Methylation<br>Methoxylation	M69-M71
	Methoxylation<br>Decarbonylation	M12-M13
	Methoxylation<br>Chalcone Formation	M54-M60
	Chalcone Formation<br>Di-Oxidation	M66

	Chalcone Formation<br>Oxidation		M34-M37
	Glucuronid Conjugation	Oxidation	M80-M81
	Glucuronid Conjugation<br>Methoxylation		M83-M86
	Glucuronide Conjugation<br>Flavanone Formation		M79
	Glucuronide Conjugation<br>Demethylation		M77
Tertiary primary	Flavanone Formation<br>Demethylation<br>Oxidation		M16-M19
	Flavanone Formation<br>Methoxylation<br>Sulfate-Conjugation		M74-M76
	Di-methoxylation<br>Decarbonylation		M61-M65
	Glucuronide Conjugation<br>Flavanone Formation<br>Oxidation		M82

© 2019 by the authors. Submitted for possible open access publication under the terms and conditions of the Creative Commons Attribution (CC BY) license (http://creativecommons.org/licenses/by/4.0/).
